# Susceptibility to COVID-19 in Pregnancy, Labor, and Postpartum Period: Immune System, Vertical Transmission, and Breastfeeding

**DOI:** 10.3389/fgwh.2021.602572

**Published:** 2021-02-17

**Authors:** Adson José Martins Vale, Amélia Carolina Lopes Fernandes, Fausto Pierdoná Guzen, Francisco Irochima Pinheiro, Eduardo Pereira de Azevedo, Ricardo Ney Cobucci

**Affiliations:** ^1^Tocogynecology Department, Medical School, Universidade Federal do Rio Grande do Norte (UFRN), Natal, Brazil; ^2^Graduate Program of Biotechnology, Laureate International Universities - Universidade Potiguar (UnP), Natal, Brazil; ^3^Medical School, Laureate International Universities - Universidade Potiguar (UnP), Natal, Brazil; ^4^Nurse Department, Nurse School, Universidade do Estado do Rio Grande do Norte (UERN), Mossoró, Brazil

**Keywords:** COVID-19, pregnancy, immunity, vertical infection transmission, breastfeeding

## Abstract

The new coronavirus (SARS-Cov-2) was first identified in late 2019 as the new RNA virus in the coronaviridae family responsible for causing COVID-19 in the residents of China's Hubei province. In mid-March 2020 WHO declared the pandemic caused by this virus as a result of thousands of people infected all over the world. Epidemiological evidence obtained from other pandemics, such as influenza and ebola, suggest that pregnant women are more susceptible to serious complications and death from viral infection. Physiological changes in the anatomical structure of the respiratory system as well as in the immune system during the pregnancy-puerperal period seem to contribute to this greater risk. Thus, pregnant women are more susceptible to be infected by the SARS-COV-2 or other viruses and to have serious COVID-19 disease. In fact, COVID-19 can alter immune responses at the maternal-fetal interface, affecting the well-being of both mother and her fetus. There is still no sufficient evidence in the literature to support the occurrence of vertical transmission and through breastfeeding, but the prevalence of prematurity was high among pregnant women infected by SARS-Cov-2. In this review, the changes in the immune system that may increase susceptibility to SARS-Cov-2 are discussed as well as the possible mechanisms involved in the transmission of the virus to the fetus by vertical transmission and during breastfeeding.

## Introduction

The disease caused by the new coronavirus (COVID-19) is currently the most serious public health problem that the world has faced (1). According to a recent report from the World Health Organization, until August 13th of 2020, 20,439,814 cases of COVID-19 have been registered with 744,385 deaths (2).

**Figure 1:**
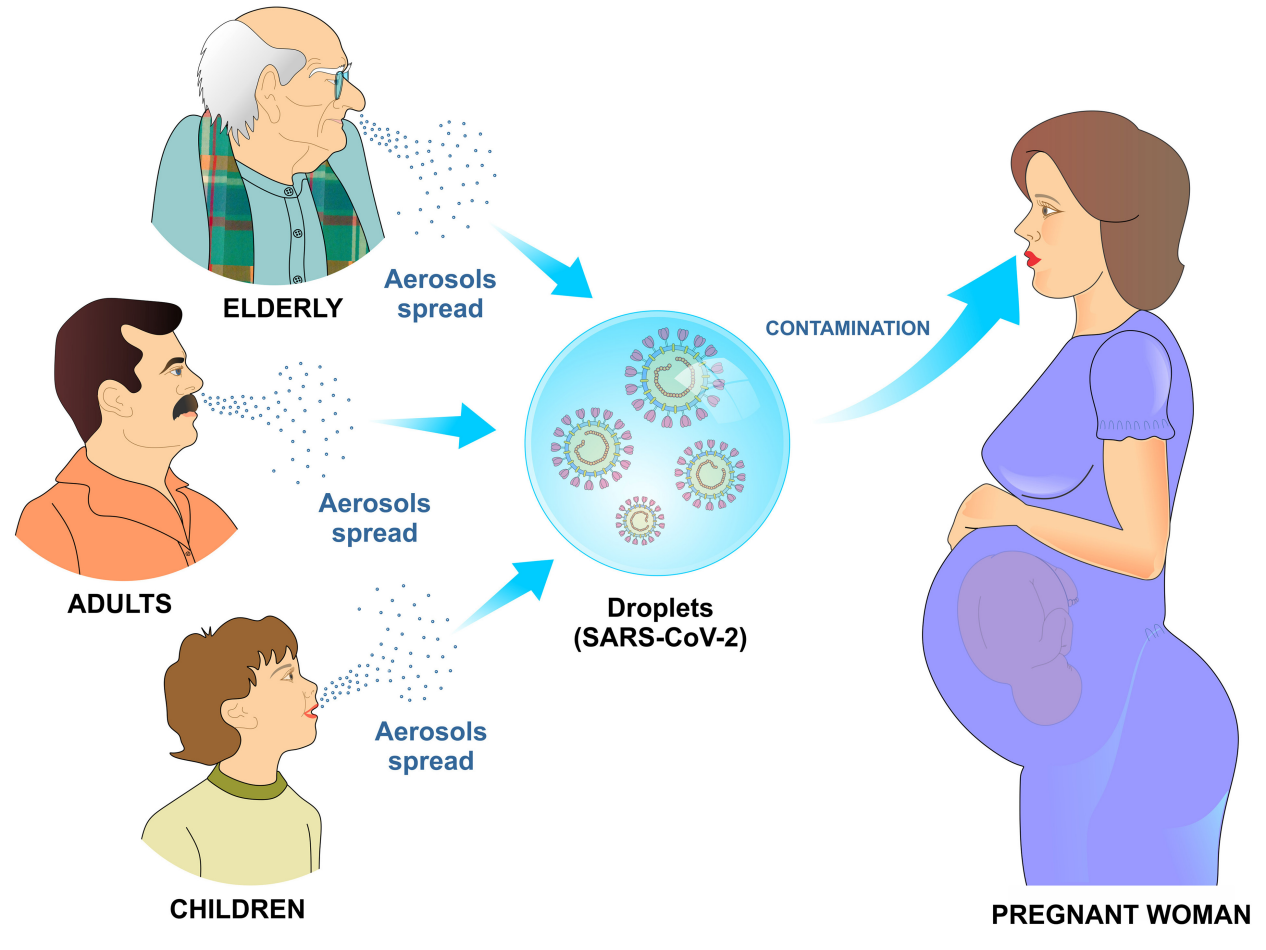
Transmission mechanism of SARS-CoV-2 by air. Infected children, adults, and elderly disseminate viral particles by sneezing or coughing. Upon reaching the mucosa of the upper airways or alveoli, the SARS-CoV-2 viral particles bind to specific receptors on the cell surface and initiate the process of cell penetration and subsequent viral replication.

SARS-Cov-2 can infect newborns, children, young adults, pregnant women and elderly (3). This virus is more contagious than the coronavirus that causes severe respiratory distress syndrome (SARS), which had infected ~8,000 people and caused 800 deaths so far. The combination of inadequate immune response and high infectivity can contribute to the SARS-CoV-2 widespread. Contagion occurs mainly through droplets and aerosols spread in the environment by the infected people (1) (Figure 1).

Once in contact with the body, SARS-CoV-2 binds to a cell surface receptor, invades the endosome and eventually fuses viral and lysosomal membranes. In mature viruses, the spike protein is present as a trimer, with three S1 receptor-binding heads, sitting on top of an S2 membrane fusion rod. Like SARS-CoV, SARS-CoV-2 recognizes the angiotensin-converting enzyme 2 (ACE2) as its receptor (4).

During pregnancy, the maternal immune system faces some challenges which includes establishing and maintaining tolerance to the fetus, as well as preserving the ability to fight against viruses and bacteria, therefore, a healthy pregnancy depends on immune adaptations. In fact, the maternal immunological system adapts and changes with the growth and development of the fetus at the different stages of pregnancy, which goes from a pro-inflammatory state (beneficial for embryo implantation and placentation) in the first trimester to an anti-inflammatory state (useful for fetal growth) in the second trimester. In the third trimester, it reaches a second pro-inflammatory state (in preparation for the start of childbirth) (3, 5).

**Figure 2:**
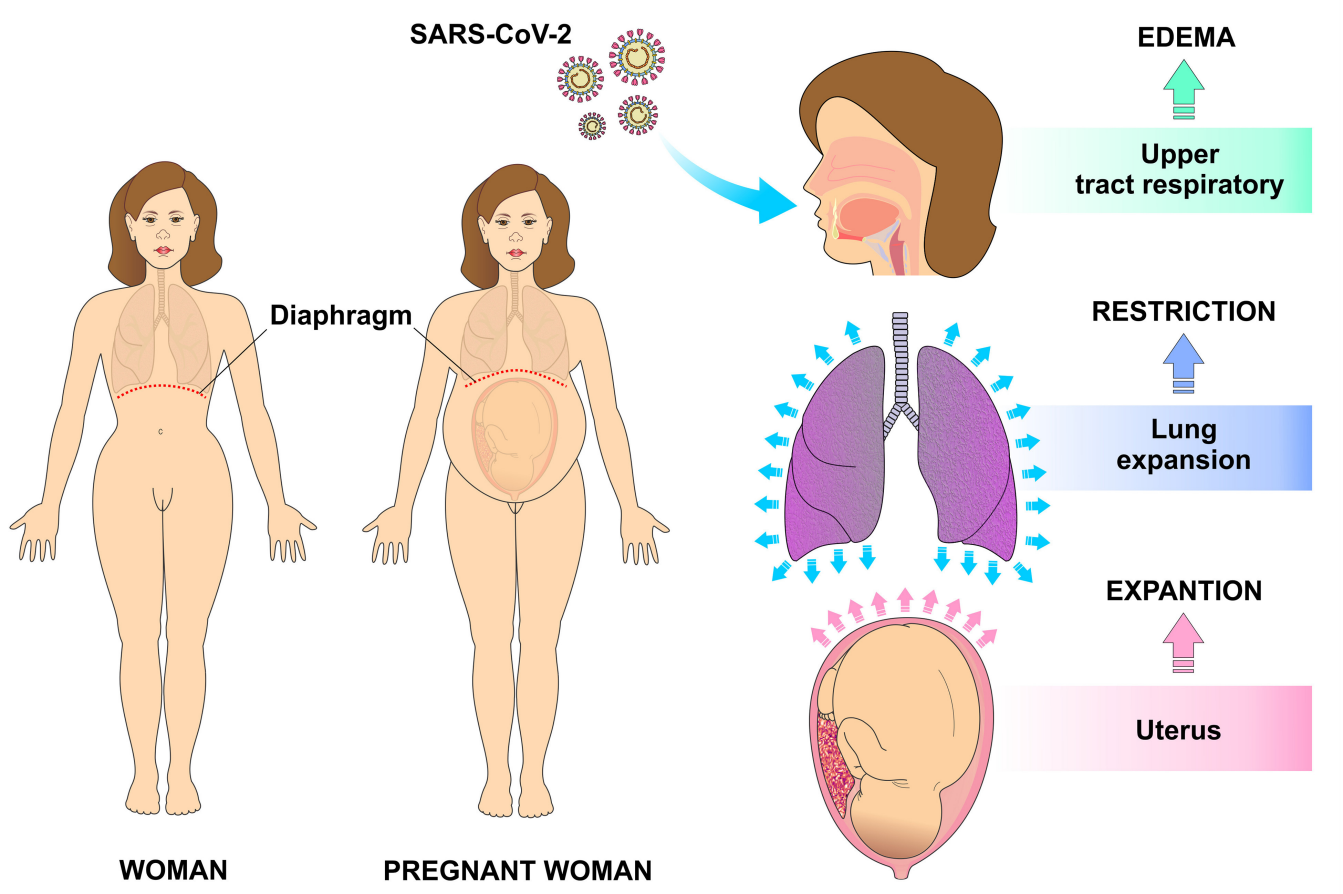
Physiological changes in the respiratory system during pregnancy that make it more vulnerable for infection. The high levels of estrogen and progesterone result in edema of the upper respiratory tract also contribute to higher risk of infections. With uterine expansion, the diaphragm is displaced superiorly, making pulmonary expansion difficult and therefore decreasing the respiratory reserve capacity.

The immune system of a pregnant woman is well-prepared to defend against the invasion of pathogens in such a way that innate immune cells like NK cells and monocytes respond more strongly to viral challenges. On the other hand, some adaptive immune responses are negatively regulated during pregnancy. In addition, the high levels of estrogen and progesterone induce the upper part of the respiratory tract to swell which, in addition to the restricted lung expansion on the last gestational trimester, make the pregnant woman more susceptible to respiratory pathogens such as SARS-CoV-2 (3, 5) (Figure 2).

Previous reports have shown that SARS infection during pregnancy can lead to premature birth, intrauterine growth restriction and spontaneous abortion. However, there is still no strong evidence of vertical transmission of SARS-Cov-2. Therefore, it seems that these complications are caused by the direct effect of this virus on mothers. Although current evidence is limited regarding the transmission of the new coronavirus during pregnancy and lactation, the potential risk of vertical transmission must not be rule out (3, 6, 7).

In this review, the main changes in the immune system that occur during pregnancy, which may increase susceptibility to SARS-Cov-2 infection, are discussed as well as the possible mechanisms involved in the transmission of the virus to the fetus by vertical transmission and during breastfeeding.

## SARS-COV-2: Mechanisms of Cell Infection and Immune Response

Coronaviruses infect host cells through protein-mediated fusion on their surface (spike protein—S). Although unusual, the spike protein can be activated by furins, which are proteases with high expression levels (8). The genomes of coronaviruses evolve through gains or losses of genes. Such genes have high plasticity, which means that the longer the genome is the greater the probabilities of adaptive mutation are, thus generating high diversity for the spike protein to change and adapt to other cell receptors (9).

**Figure 3:**
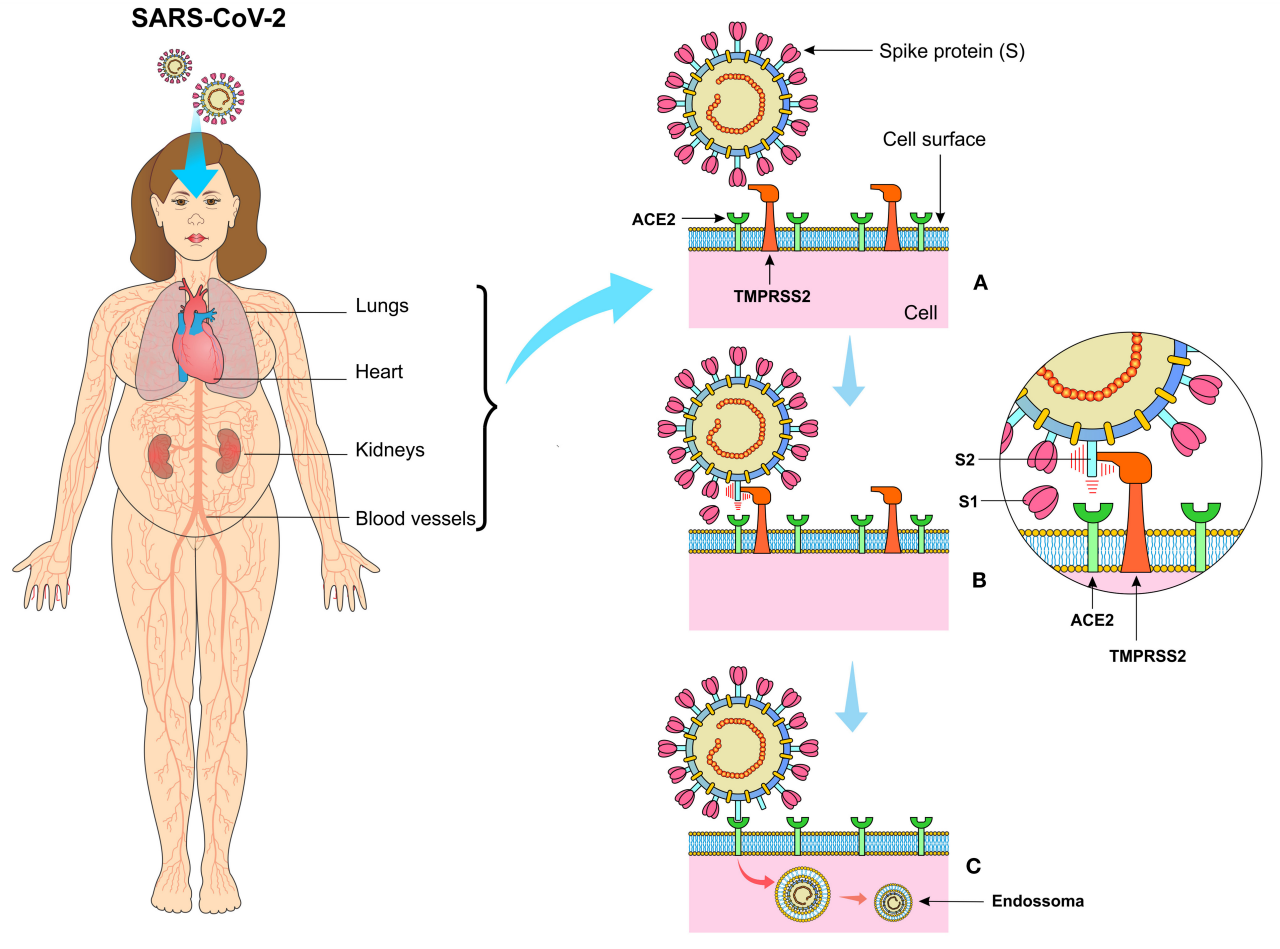
**(A)** The SARS-CoV-2 virus presents the spike protein (S) on its surface, expressed in the form of a spike that binds to angiotensin-converting enzyme 2 (ACE2) present on the surface of cells especially in the lungs, kidneys, heart, vessels, and adipose tissue. **(B)** Transmembrane Serine Protease 2 (TMPRSS2) cleaves protein S in units S1 and S2, making it possible for the virus to bind to ACE2. **(C)** The viral particle is enclosed by the cell membrane creating the endosome, which after the proteolytic action of the structural components of the virus, the viral RNA is released in the cytoplasm of the infected cell.

The horseshoe bats work as natural hosts and are reservoirs for SARS-CoV (10). Since the first step in the viral replication cycle is mediated by protein S, it offers several potential therapeutic targets. Protein S uses the angiotensin-2 converting enzyme (ACE2) and sialic acids linked to gangliosides on the cell surface to enter the cell (11). Therefore, cell penetration by coronaviruses requires the activation of protein S by cellular proteases, which affects the cleavage of protein S, thus allowing the fusion of viral and cellular membranes. The SARS-S receptor becomes involved with ACE2 as an input receptor (12) and releases the cellular serine protease TMPRSS2 to activate the S protein (13, 14) (Figure 3).

Even though SARS-S and SARS-2-S share 76% of amino acid identity, no study has shown how SARS-2-S or SARS-S uses ACE2 and TMPRSS2 to adhere to the target cell (15, 16). In this context, the transmembrane serine protease TMPRSS2 activates the coronavirus peak protein (17). Since other coronaviruses use ACE2 as a cell receptor, it seems that host factors other than ACE2 may contribute to the highly efficient zoonotic transmission of SARS-CoV-2 from person to person (18, 19). In addition, it has been evidenced a lower expression of cytokines and chemokines in mice deficient in TMPRSS2 in comparison to those that had TMPRSS2 activity after coronavirus infection. Viral replication is probably one of the main causes of the high levels of inflammatory chemokines observed in mice, even though the TMPRSS2 involvement in the inflammatory reactions has also been evaluated. Thus, activation of coronavirus S proteins by target cell proteases are essential for viral entry into the cells and encompasses protein S cleavage at S1/S2 and S2 sites. The S1/S2 cleavage site of SARS-2-S houses several arginine residues, which indicates high cleavage activity (20).

**Figure 4:**
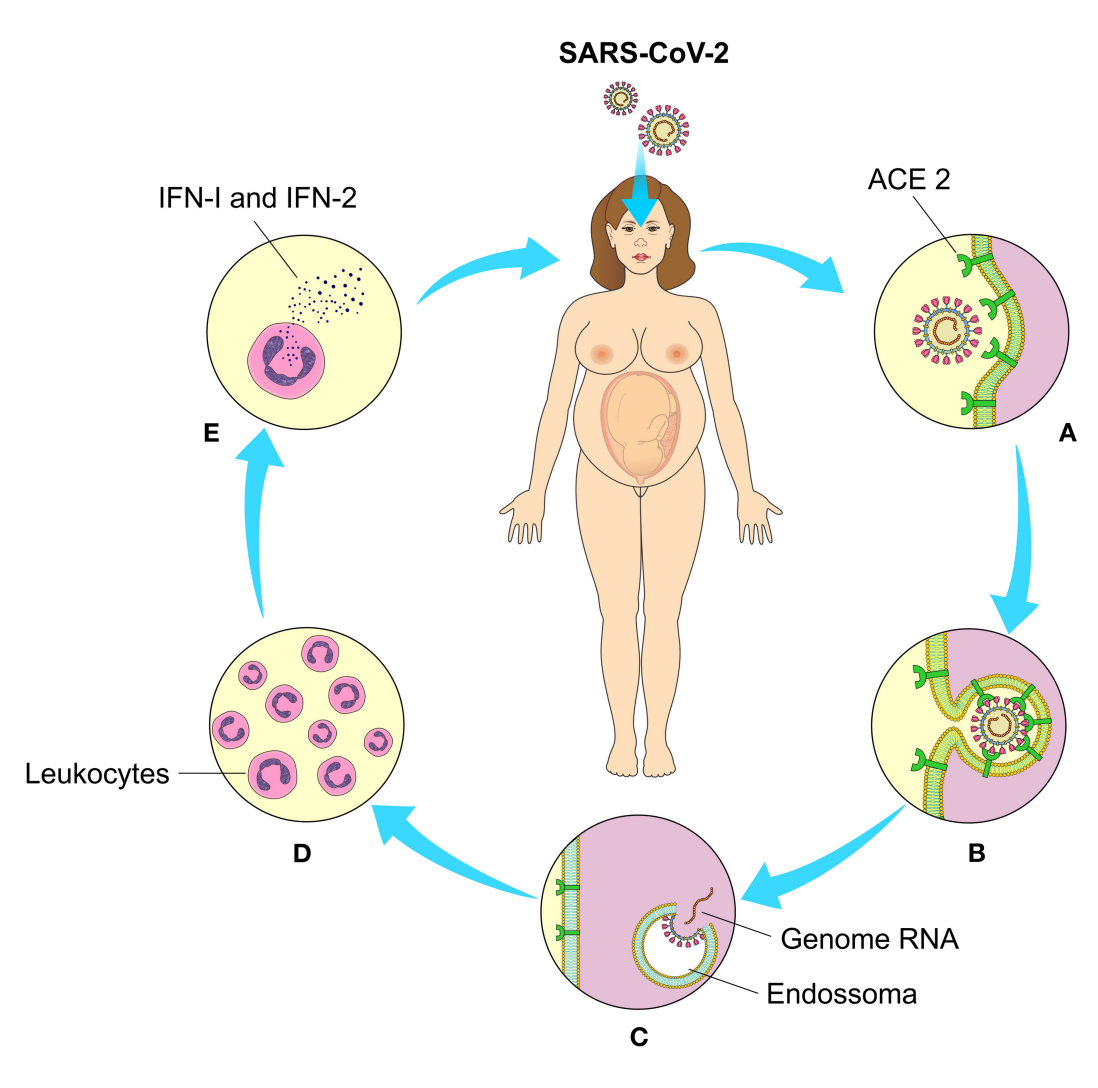
Cell binding, interiorization, and cellular response to SARS-CoV-2. **(A)** Through their binding to angiotensin 2-converting enzyme (ACE2) on the cell's surface, **(B)** the viral penetrates inside the cells and **(C)** after the enzymatic action of the endosomes release their genetic material (RNA) for the production of the structural components of new viral particles. **(D)** Intracellular receptors detect viral RNA replication and mediate leukocyte chemotaxis and **(E)** interferon production (IFN-I and II).

The physiological immune response against SARS-CoV-2 is usually initiated at the cellular level after viral replication. Cellular detection is mediated by a family of intracellular receptors that detects aberrant RNA structures that usually form during virus replication. Initially, there is an engagement of cellular antiviral defenses, mediated by the transcriptional induction of type I and III interferons (IFN-I and IFN-III), followed by a sub-regulation of IFN-stimulated genes (ISGs). Antiviral response also involves recruitment and coordination of specific subsets of leukocytes, orchestrated mainly by the secretion of chemokines (21). Immune responses play an essential role in determining the progression of SARS-CoV-2 infection, as damaged lung cells induce a local immune response, which recruits macrophages and monocytes to respond to infection (22) (Figure 4).

**Figure 5:**
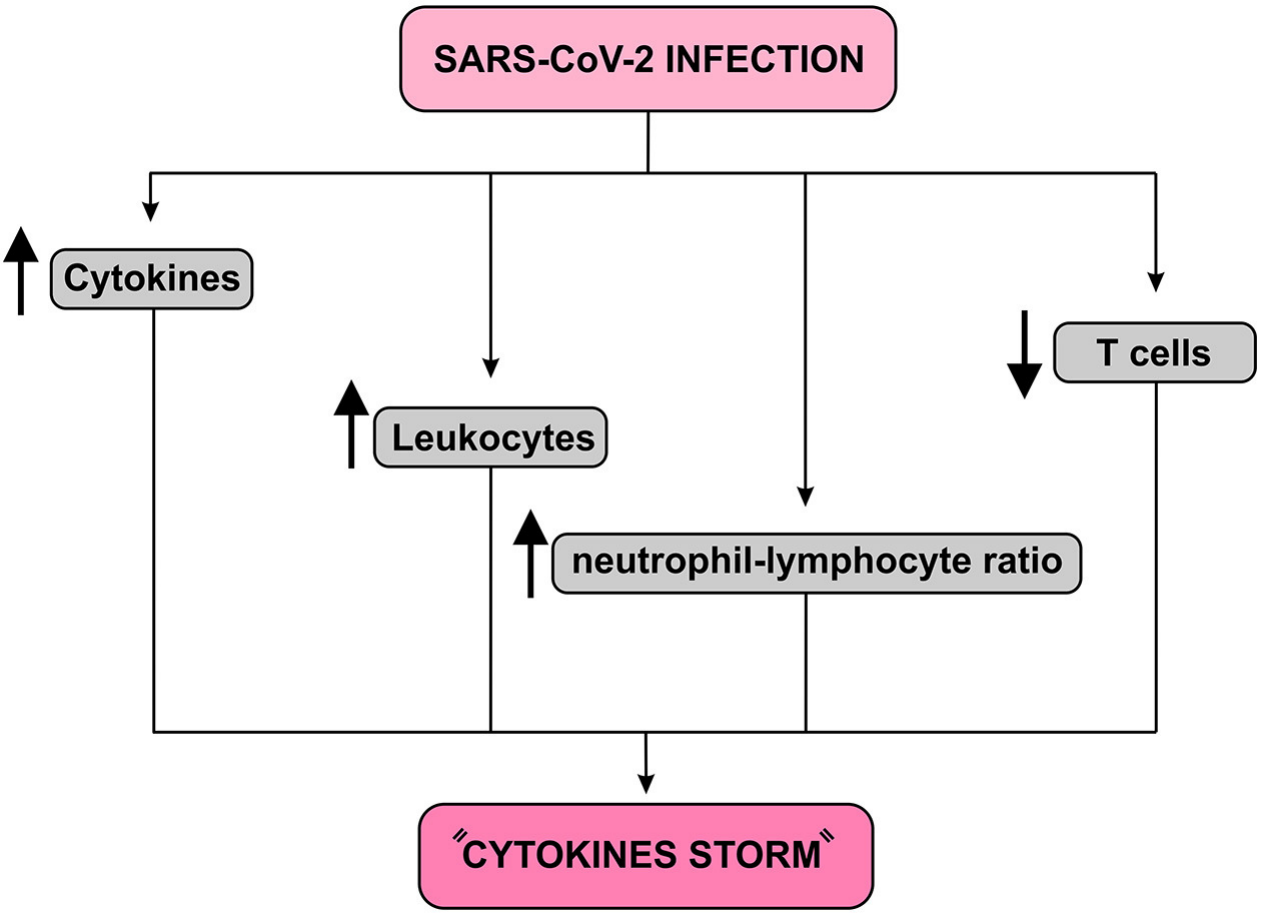
Immune response to SARS-CoV-2 infection diagram. This infection causes a fall in T cells and an increase in leukocyte count and neutrophil-lymphocyte ratio. In addition, it increases the release of cytokines resulting in a condition known as “Cytokines storm,” which may lead to pulmonary edema, severe hypoxia, respiratory failure and, finally, multiple organ failure.

The relocation of NK cells, macrophages and plasmacytoid dendritic cells (pDC) to the lungs has been associated with increased levels of cytokines and chemokines. In fact, dysregulation of immune responses commonly occurs in severely affected patients, which includes excessive secretion of inflammatory cytokines and imbalances in the proportion of naive helper T cells, memory helper T cells and regulatory T cells. SARS-CoV-2 can induce dysregulation of immune responses in susceptible individuals, as demonstrated by the decrease in lymphocytes, especially T cells, increased leukocyte count and neutrophil-lymphocyte ratio as well as other imbalances in the immune cell population. In addition, severely affected patients are accompanied by a significant increase in the proportion of naive T helper cells as well as by a reduction in memory T helper cells and regulatory T cells (23, 24) (Figure 5).

A recent study involving Chinese patients with SARS-CoV-2 showed high plasma concentrations of IL-1B, IL-1RA, IL-7, IL-8, IL-9, IL-10, basic FGF, GCSF, GMCSF, IFN-γ, IFN-γ (IP)−10-induced protein, monocyte chemotactic protein 1 (MCP1), MIP1A, MIP1B, and TNF-α. The authors reported significant overproduction of IL-2, IL-7, IL-10, GCSF, IP-10, MCP1, MIP1A, and TNF-α. Deregulation of cytokine levels has been demonstrated in almost all patients, but clear differences have been reported in the levels of various cytokines between severely affected patients and those with moderate or mild symptoms (20). These massive cytokine outbreaks result in a severe immunopathological condition known as “cytokine storm,” which can lead to several pathological consequences including extensive pulmonary edema, acute respiratory distress syndrome and multiple organ failure (24, 25).

Severe and lethal cases of SARS-CoV and MERS-CoV manifest with greater accumulation of neutrophils and monocytes-macrophages in the lungs. In fact, this was the main mechanism involved in lung damage in both viral infections. It has been hypothesized that in SARS-CoV and MERS-CoV infections the delay in the IFN type I response compromised the control of viral replication. This, in turn, would lead to an increase in the influx of neutrophils and monocyte-macrophages (26). Increased accumulation and persistent activation of these cells would cause lung damage with clinical manifestations that include pneumonia and severe respiratory distress syndrome (27).

Like many other pathogens, SARS-CoV-2 develops mechanisms that help to evade the host's immune system. One of these mechanism is the persistent activation of the NLRP3 inflammasome (NACHT, LRR, and PYD domains containing protein 3), which is a component of the innate immune system that induces the activity of caspase-1 and pro-inflammatory cytokines such as interleukin (IL)−1β and secretion of IL-18 in macrophages (28).

Recent reports have shown that lymphopenia was a frequent finding in most patients with COVID-19 who required hospitalization, which might be due to the migration of T cells to the lungs (27). Such clinical findings were evidenced by chest radiographs and lung computed tomography (29, 30). This migration of lymphocytes accompanied by macrophages, causes interstitial damage that impairs the gas exchange and therefore, compromises oxygenation. This is why hypoxemia and dyspnea are two of the main predominant characteristics of COVID-19 infected patients. Therefore, the development of the respiratory distress syndrome observed in these patients may be a reflection of these clinical conditions (30).

The lungs of COVID-19 infected patients exhibit characteristics consistent with a non-specific inflammatory response, such as intense infiltration and edema (29, 30). Other characteristics found in these patients include thickening and damage of the alveolar septa, severe desquamation of alveolar epithelial cells and infiltration of the alveolar space. This intense inflammatory process eventually leads to necrosis, infiltration, and hyperplasia (31, 32).

## Pregnancy, Immunology, and Susceptibility to SARS-COV-2

Human decidua during pregnancy involves a high number of immune cells, predominantly macrophages, natural killer (NK) cells, and regulatory T cells (Treg). During the first trimester of pregnancy, macrophages and natural killer (NK) cells accumulate around the trophoblastic cells, which results in a protective effect, preventing abortion of the allogeneic fetus (33, 34). The maternal immune system protects the mother from aggressors coming from the environment and prevents damage to the fetus. On the other hand, the fetus activates the immune response that changes the way the pregnant woman responds to the environment, which makes the immune response very unique during pregnancy. Therefore, this particular immune system must be characterized by a modulated immune condition, rather than a suppressed one (33).

In pregnancy, progesterone has immunomodulatory properties that in addition to preventing the mother from recognizing the fetus as an antigen, it can influence the evolution of autoimmune diseases with improvement in conditions such as rheumatoid arthritis. During pregnancy, there is an increase in anti-inflammatory molecules such as interleukin-1 receptor antagonist (IL-RA) and tumor necrosis factor-α receptor (TNF-R), whereas a decrease in IL-1b and tumor necrosis factor-α (TNF-α) are observed (35). In the human placenta, the trophoblast expresses pattern recognition receptors (PRRs) that act as sensors to detect external aggressors. Through them, the trophoblast is able to recognize bacteria and viruses, and then secrete cytokines and interferons. Interferons are potent antiviral proteins that also have important immunomodulatory functions (34, 36). In addition, active transport of antibodies of the IgG class produced by maternal humoral immunity occurs through the placenta after 16 weeks of pregnancy, resulting in increased fetal immunity against microorganisms (34).

**Figure 6:**
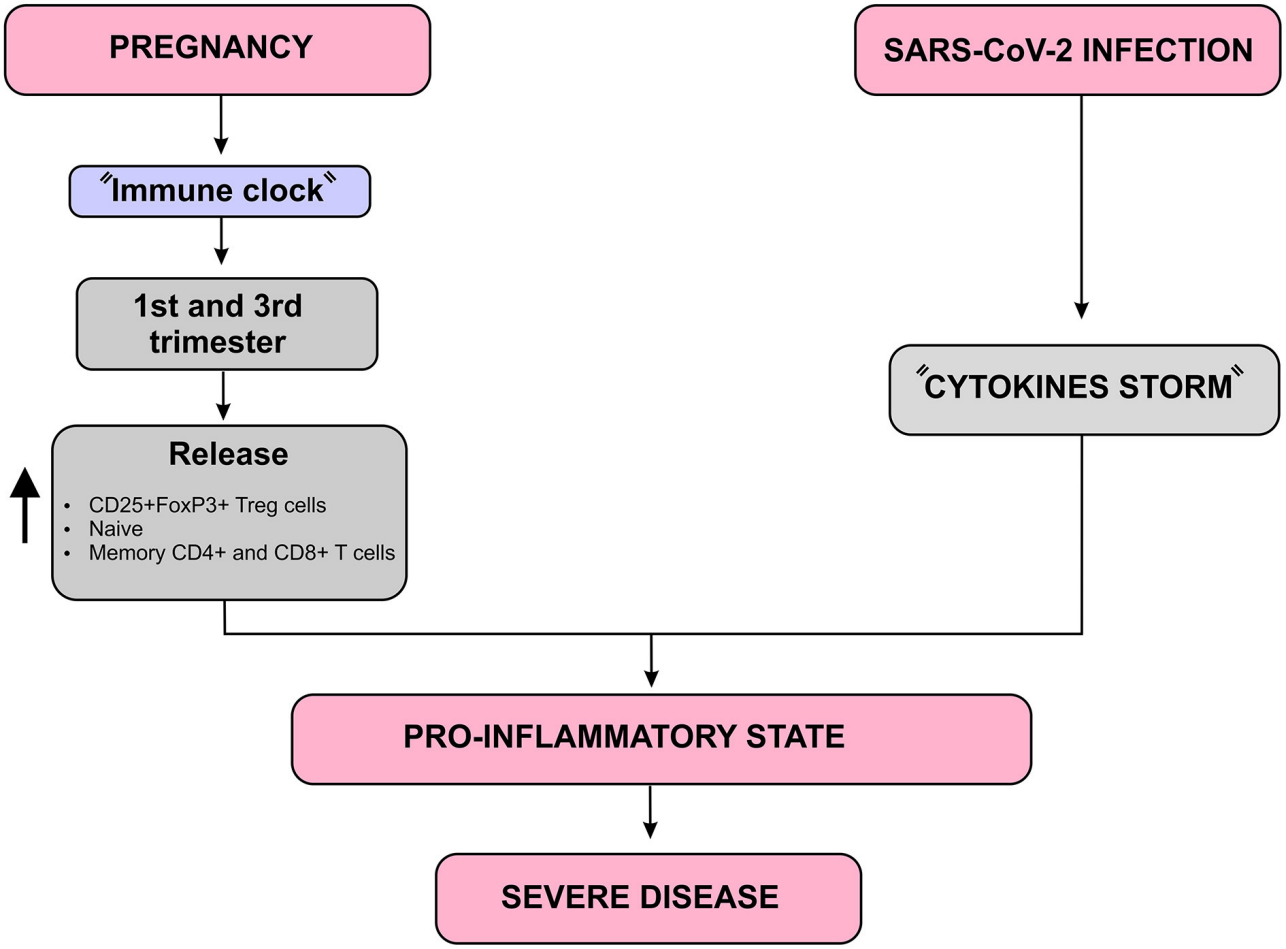
Immunological complications that may worsen the SARS-CoV-2 infection prognosis in pregnant women diagram. In addition to the “Cytokines storm” induced by SARS-CoV-2, there is a natural increase in pro-inflammatory mediators such as CD25 + FoxP3 + Treg cells, naive and memory CD4 + and CD8 + T cells during the first and third trimesters of pregnancy, which is known as “Immune clock.” These two aspects together lead to a more severe pro-inflammatory state which may worsen the SARS-CoV-2 infection, especially during the first and third trimesters of pregnancy.

Immunity undergoes some changes during pregnancy that avoids an exacerbated immunological response against the allogeneic fetus, but maintains an adequate immune response against invading microorganisms (3). Aghaeepour et al. described how a “immune clock” occurs during pregnancy through a progressive increase in the release of CD25+FoxP3+ Treg cells, naive and memory CD4+ and CD8+ T cells as well as γδ T cells (37). Considering that pregnant women are in a pro-inflammatory state in the first and third trimester, the SARS-CoV-2-induced cytokine storm may result in a more severe inflammatory state in these women. In addition, the occurrence of maternal inflammation as a result of viral infections during pregnancy can affect various aspects of the fetal brain and can lead to a wide range of neuronal dysfunctions and behavioral phenotypes (7) (Figure 6).

However, changes in the levels of estrogen and progesterone from the first gestational trimester cause respiratory, cardiovascular and immune changes that make pregnant women more susceptible to SARS-Cov-2 infection, in addition to an increased risk of developing severe acute respiratory syndrome (SARS). The effect of progesterone on the nasal mucosa facilitates the adhesion of the virus and hinders its elimination. Moreover, the increase in oxygen consumption due to vascular congestion and the decrease in the functional residual capacity of the lung contribute to an increased risk for severe respiratory symptoms in infected pregnant women (38).

**Figure 7:**
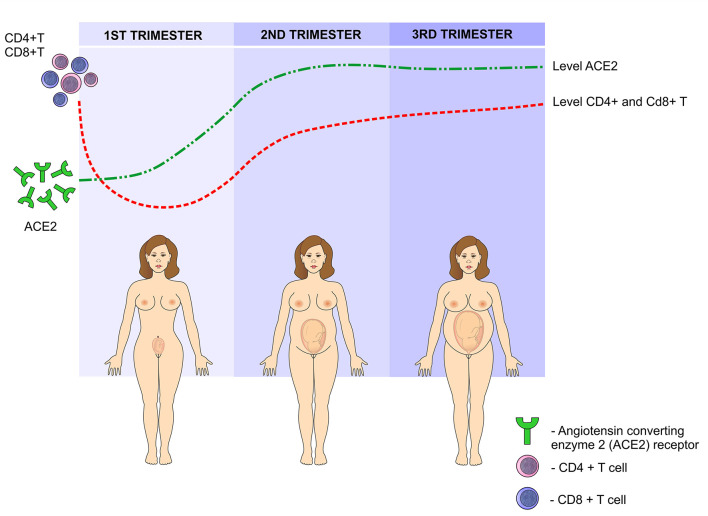
SARS-CoV-2 infection during pregnancy: In the first trimester of pregnancy, the levels of angiotensin-converting enzyme 2 (ACE2) increase, peaking in the second trimester and reaching a plateau in the third semester. This enzyme facilitates the binding of the virus to the cells. On the other hand, due to the presence of paternal antigens foreign to the mother's body, immunity is modulated by reducing the levels of CD4 + T and CD8 + T lymphocytes in the first trimester, gradually increasing in the second and third trimesters.

Such changes in the levels of estrogen and progesterone in the first trimester cause a reversible degeneration in the thymus, with a decrease in CD4 + and CD8 + T cells. In addition, the activity of these cells significantly reduces, contributing to a greater susceptibility to infections during pregnancy (38). Another risk factor involves the angiotensin converting enzyme 2 (ACE2) receptor, to which the virus binds before infecting the cell and it is upregulated during pregnancy. As a result of higher ACE2 expression, pregnant women may be at an elevated risk of complications from SARS-CoV-2 infection (39). Previous studies have reported an increase in these receptors in the kidneys of pregnant women, which may contribute to the efficient regulation of blood pressure during pregnancy. However, it can favor the binding of the virus and therefore, facilitating its entry into the host's cells (38) (Figure 7).

## SARS-COV-2 in Pregnancy, Childbirth, and its Vertical Transmission

Despite the low rates of morbidity and mortality from SARS-CoV-2 infection in children and women of reproductive age, these groups can be disproportionately affected by the collapse of health care services, especially in developing countries, with a possibility of increasing the prevalence of maternal mortality up to 38.6% in the worst case scenario as a consequence of this pandemia (40). A study involving 978 cases of pregnant and postpartum women infected by SARS-Cov-2 in Brazil revealed that 207 (21.2%) were admitted to the intensive care unit and among them 124 died. The authors pointed out that the high mortality due to COVID-19 in that country can be explained by the low quality of prenatal care, the insufficient resources for the management of critical patients in emergencies and the barriers imposed by the pandemic for access to the public health system (41). A recent report revealed that maternal mortality in Brazil was much higher than that in Iran, Mexico, United Kingdom, France and United States, in which 7, 5, 1, and 16 deaths by Covid-19 have been recorded during pregnancy and puerperium, respectively (42).

**Figure 8:**
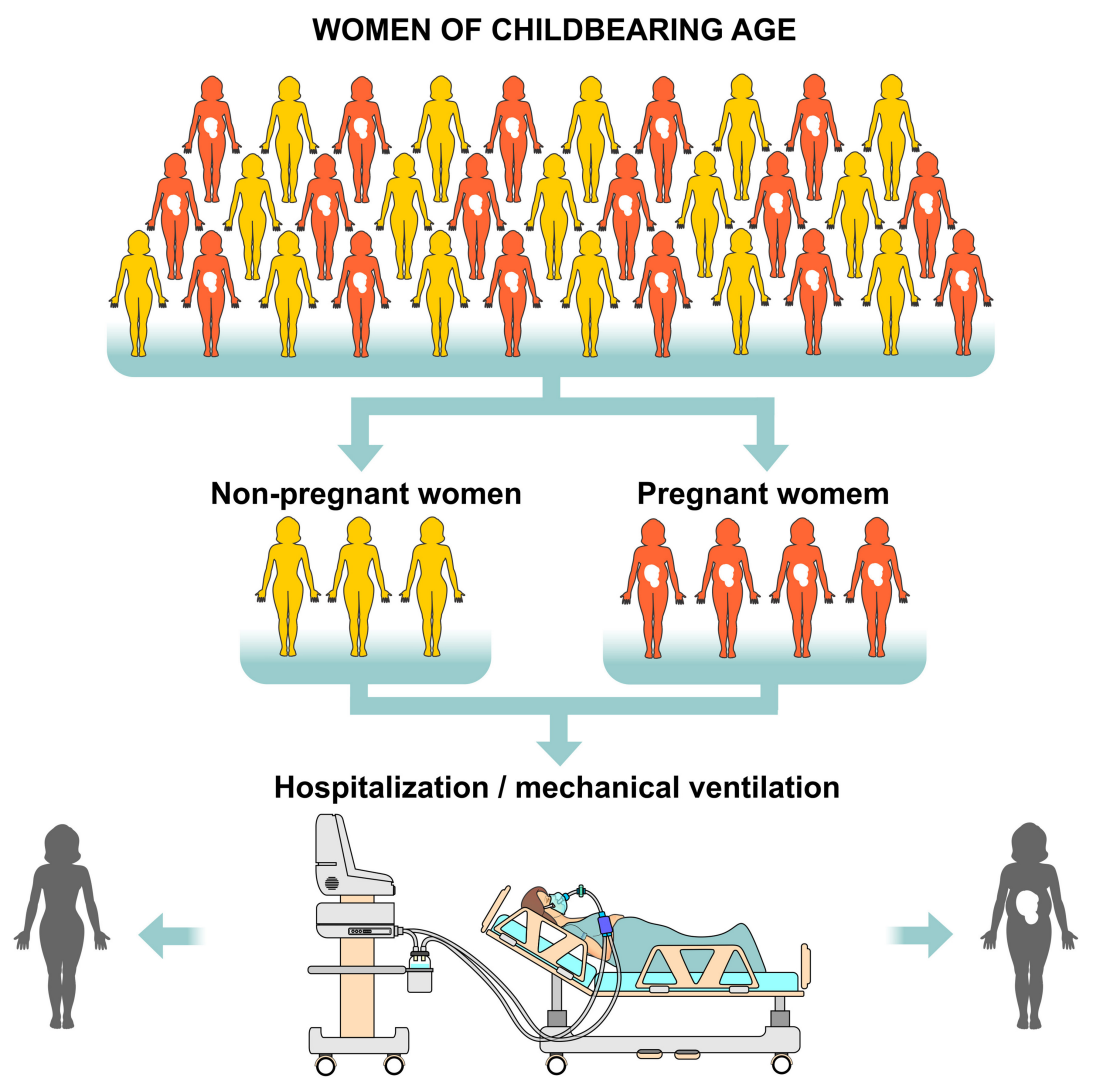
Risk of hospitalization and need for mechanical ventilation in women of childbearing age with SARS-CoV-2. Considering all women of childbearing age (pregnant and non-pregnant) who are positive RT-PCR (Reverse Transcription Polymerase Chain Reaction) for the virus, pregnant women are at greater risk of being hospitalized and requiring mechanical ventilation. However, the mortality rate is identical in both pregnant and non-pregnant groups.

The physiological consequences of SARS-CoV-2 infection in pregnancy, especially in the cardiovascular and respiratory systems, are a result of the high levels of estrogen and progesterone, as well as immunological suppression and increased blood volume, heart rate, oxygen consumption and uterine volume. The upper respiratory tract tends to swell and the lung expansion is restricted with the progression of pregnancy, which may increase the susceptibility to respiratory infections and therefore, a greater need for intensive care and mechanical ventilation occurs during pregnancy in case of a respiratory virus infection (43). Data published by the Center for Disease Control (CDC) revealed that 31% of 8,207 pregnant women with SARS-CoV-2 infection needed hospitalized and after adjusting for age, race and comorbidities, pregnant women had a significantly higher risk than the other women admitted to the intensive care unit needing mechanical ventilation. However, mortality rate (0.2%) in pregnant women aged from 15 to 44 years old was identical to that found in non-pregnant women (44) (Figure 8).

In order to prevent high maternal and neonatal morbidity and mortality, the International Federation of Gynecology and Obstetrics (FIGO) has published recommendations on the four main aspects of pregnant and postpartum women care that have been infected with SARS-CoV-2: outpatient prenatal care, care in obstetric screening centers, intrapartum care and post-natal care, childbirth and neonatal care. However, before the pandemic, access to services specialized in maternal and child care was already precarious in many countries, which has worsened with the increased demand for intensive care beds due to COVID-19 (41, 45).

Despite the increased risk of complications due to immunological status and physiological changes in pregnancy as well as IFGO recommendations, data from seven systematic reviews (46–52) about COVID-19 in pregnant women point out that the prevalence of severe respiratory distress syndrome is not different from that found in the infected population and that the mortality is equally low during pregnancy and childbirth. These reviews included 637, 538, 385, 324, 136, 92, and 51 pregnant women infected with SARS-CoV-2. All these studies showed that the most common signs and symptoms were fever and cough, with the minority presenting severe respiratory distress syndrome and requiring intensive care as well mechanical ventilation. The prevalence of cesarean sections varied between 69.4 and 84.7% in the studies, with the most common maternal complication being preterm delivery and a 1.6% of maternal mortality rate as reported in the study conducted with the largest number of pregnant women, while the others reported none or just one death. Turan et al. (51) concluded that maternal age, obesity, diabetes mellitus, and elevated D-dimer and interleukin-6 are predictive of poor pregnancy outcomes in those with COVID-19.

These results are similar to those reported by a prospective population-based study involving 194 maternity hospitals in the United Kingdom with 427 infected pregnant women in which only 10% needed ventilatory support and five (1%) died from Covid-19 (53). Another study conducted in New York revealed that among 70 pregnant women whose viral RNA was detected by RT-PCR, 55 (78%) were asymptomatic. The most common complaints among symptomatic women were cough and fever with only 3 presenting hypoxia, whereas none required mechanical ventilation and only one was admitted to the intensive care unit without any death (54).

SARS-CoV-2 can be identified by RT-PCR and serological tests from upper airway smears and blood from pregnant women and newborns. Differentiating the vertical transmission of the virus from contamination in the neonatal period is of paramount importance, but the data in the literature are still controversial about the occurrence of intrauterine infection (55).

RT-PCR has been the most frequently used method for diagnosis of COVID-19 in pregnant women and newborn, as shown by several systematic reviews that studied the vertical transmission of this virus (46–49, 51, 52). A swab collection took place on newborns on the first and second days of life and the polymerase chain reaction was positive for SARS-CoV-2 in only 1, 4, 5, and 8, respectively, with the authors concluding that there is a need for more studies that can prove if there is vertical transmission of the virus. However, in another systematic review the authors revealed that of 936 neonates born from COVID-19 infected mothers, 27 were viral RNA positive for SARS-CoV-2 (nasopharyngeal swab) and that SARS-CoV-2 viral RNA testing in neonatal cord blood was positive in 2.9% (1/34), 7.7% (2/26) of placenta samples and 9.7% (3/31) of fecal/rectal swabs, concluding that there is evidence of SARS-CoV-2 vertical transmission when the infection occurs in the third trimester of pregnancy (56).

Likewise, a systematic review investigated 50 studies involving a total of 606 neonates with the purpose of assessing evidence on vertical transmission of SARS-CoV-2 (57). The authors point out that only 20 newborns presented clear evidence of viral infection (17), where the virus was detected in 8 placentas, in 3 samples of breast milk and 1 in the amniotic fluid. Despite these findings pointing to the possibility of transmission during pregnancy, they conclude that further studies need to be conducted through analysis of the virus in larger numbers of placenta, milk and amniotic fluid.

Algarroba et al. reported a case of severe respiratory distress syndrome in a pregnant woman at 28 weeks in which it was possible to detect SARS-CoV-2 in the placental syncytiotrophoblast. However, PCR detection in amniotic fluid or placenta has not been investigated yet. In addition, swabs collected from the newborn in the second and third days of life did not report the presence of the virus (58). Facchetti et al. analyzed post-partum placentas and neonates who presented positive PCR and SARS-CoV-2 pneumonia shortly after birth and found an elevated expression of viral proteins S and N in the placental tissue. Thus, the authors claim to have provided some evidence for maternal-fetal transmission of SARS-CoV-2 (59). Likewise, Fenizia et al. analyzed through nasopharyngeal and vaginal swabs the presence of viral RNA by PCR in 31 infected pregnant women and in their respective newborns. In addition, they investigated specific anti-SARS-CoV-2 antibodies and the expression of genes involved in inflammatory responses in placenta, breast milk, amniotic fluid and in maternal and umbilical cord plasma. The authors found viral genome and antibodies against SARS-CoV-2 in samples of umbilical cord blood and breast milk, which seems to support the hypothesis of *in utero* vertical transmission of SARS-CoV-2 (60).

Sisman et al. reported a case of vaginal delivery occurring at 34 weeks of gestation in a pregnant woman with positive RT-PCR reagent for SARS-CoV-2. They state that the newborn was immediately separated from the mother after delivery, being admitted to a neonatal intensive care unit. Nasopharyngeal swabs were collected at 24 and 48 h of life in which the results were positive for SARS-CoV-2 infection and the neonate presented fever, tachypnea and 78% oxygen saturation in room air on the second day after birth. The authors performed an immunohistochemical study to detect the virus in the placenta. They further evaluated the placental tissue by electron microscopy and detected viral nucleocapsid protein and viral-like particles in the cells of the syncytiotrophoblast. However, the authors did not perform PCR in breast milk, amniotic fluid and umbilical cord blood (61).

**Figure 9:**
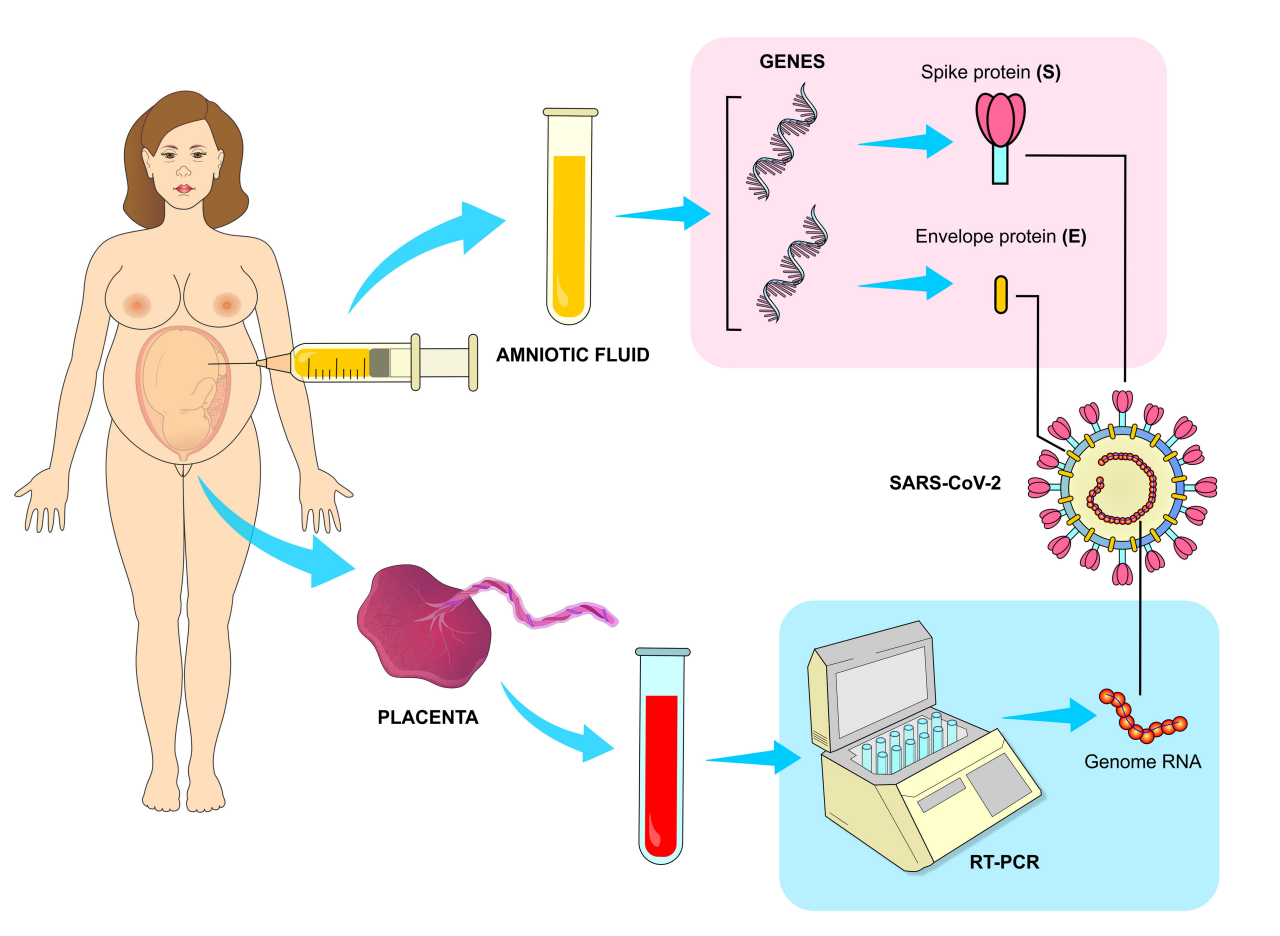
Vertical transmission of SARS-CoV-2. The transmission of the virus from infected mothers to fetus has been confirmed by RT-PCR (Reverse Transcription Polymerase Chain Reaction) method, which has detected a high viral load in the placenta. The presence of viral genes responsible for translating structural proteins E and S were also detected in samples of amniotic fluid collected during cesarean surgeries of infected mothers.

Vivanti et al. claim to have proven placental transmission of SARS-CoV-2 to a neonate who had brain injury similar to that of infected adult patients. The virus was transmitted from a mother who was infected during the last gestational trimester. In this case, placental infection was confirmed by immunohistochemistry and RT-PCR. The viral genes E and S were detected in the amniotic fluid collected during the cesarean procedure. In addition, viral RNA was detected by RT-PCR in the blood and bronchoalveolar lavage fluid of the newborn (62) (Figure 9).

In a recent study carried out in New York, swabs were collected in the first 24 h of life in 71 newborns of infected pregnant women in which no SARS-CoV-2 virus was detected by RT-PCR. Placental pathology was performed in 28 infected and 99 non-infected parturients, with a greater presence of thrombi and meconium in the placentas of women with Covid-19 (54). On the other hand, a population-based study involving 427 pregnant women with Covid-19 carried out by Knight et al. found positive RT-PCR in 12 (5%) among 265 newborns, with six being detected in the first 12 h of life (53).

A review study conducted by Lamouroux et al. reported the diagnosis of neonatal infection in 4 out of 71 newborns in the first 48 h of life. The authors pointed out that in 2 cases the PCR was negative on the sixth day of life, which is unexpected in cases of congenital infection by any pathogen. SARS-CoV-2 was detected by PCR in umbilical cord blood, amniotic fluid, placenta, vagina, and breast milk in 12, 10, 5, and 3 samples, respectively (55).

In another review (63), swabs were collected from 179 newborns from pregnant women infected in the third trimester of pregnancy with SARS-CoV-2 being detected in only six. In addition, the virus was searched in 37 samples of amniotic fluid and 48 samples of umbilical cord blood from these parturients and all were negative. Thus, the authors concluded that more evidence is needed to prove whether there is a risk of congenital COVID-19 infection.

Finally, the ideal gestational age for birth and the route of delivery must be determined by maternal conditions that can be aggravated by infection and fetal vitality. Pregnant women who have been infected in the first trimester must wait for the evolution for the delivery at term. For those infected in the third trimester and who are in the recovery phase, postponing childbirth until the mother is fully recovered seems to be the best choice. Early delivery by cesarean section is only indicated for pregnant women with severe respiratory distress syndrome, while among those who have developed mild symptoms without compromising fetal vitality, vaginal delivery is safe and recommended. Transmission of SARS-CoV-2 person-to-person in the delivery room through the healthcare team should be avoided through protective measures for both patient and staff (64).

## SARS-COV-2 in the Postpartum

**Figure 10:**
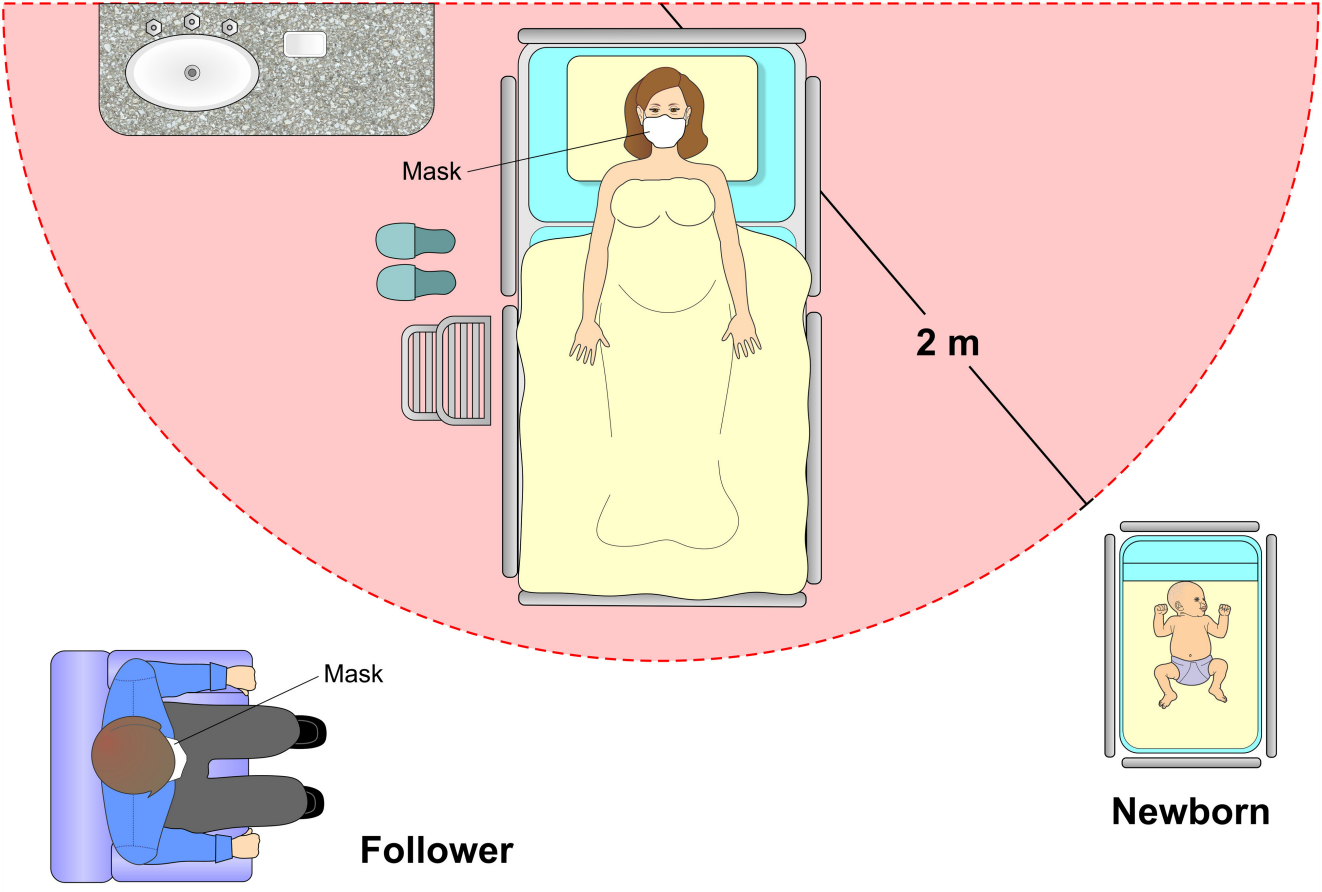
Postpartum care. If the mother is in good health condition and does not require intensive care, it is recommended that the newborn remain in an appropriate cradle at a distance of 2 meters from the mother. The puerperal woman must always remain with a protective mask with an adequate filtering index and obey strict methods of hand hygiene. The presence of a companion is also allowed, following the rigor of hand hygiene with 70% alcohol and the use of an appropriate mask.

In the immediate postpartum period, a minimum distance of two meters from the cradle to the bed of the mother infected with SARS-CoV-2 is recommended. Isolation with a screen or curtains and use of masks by both parturient and companion are also advised. However, a systematic review study including 666 neonates did not show high rates of postnatal SARS-CoV-2 infection after vaginal births, breastfeeding and mother-baby interaction (65) (Figure 10).

The RT-PCR detection of SARS-CoV-2 in umbilical cord blood has not been proven to be the best target for virus detection in both vaginal and cesarean delivery. Thus, there is no reported increased risk of vertical transmission with umbilical cord clamping between 1 and 3 min after birth (64).

**Figure 11:**
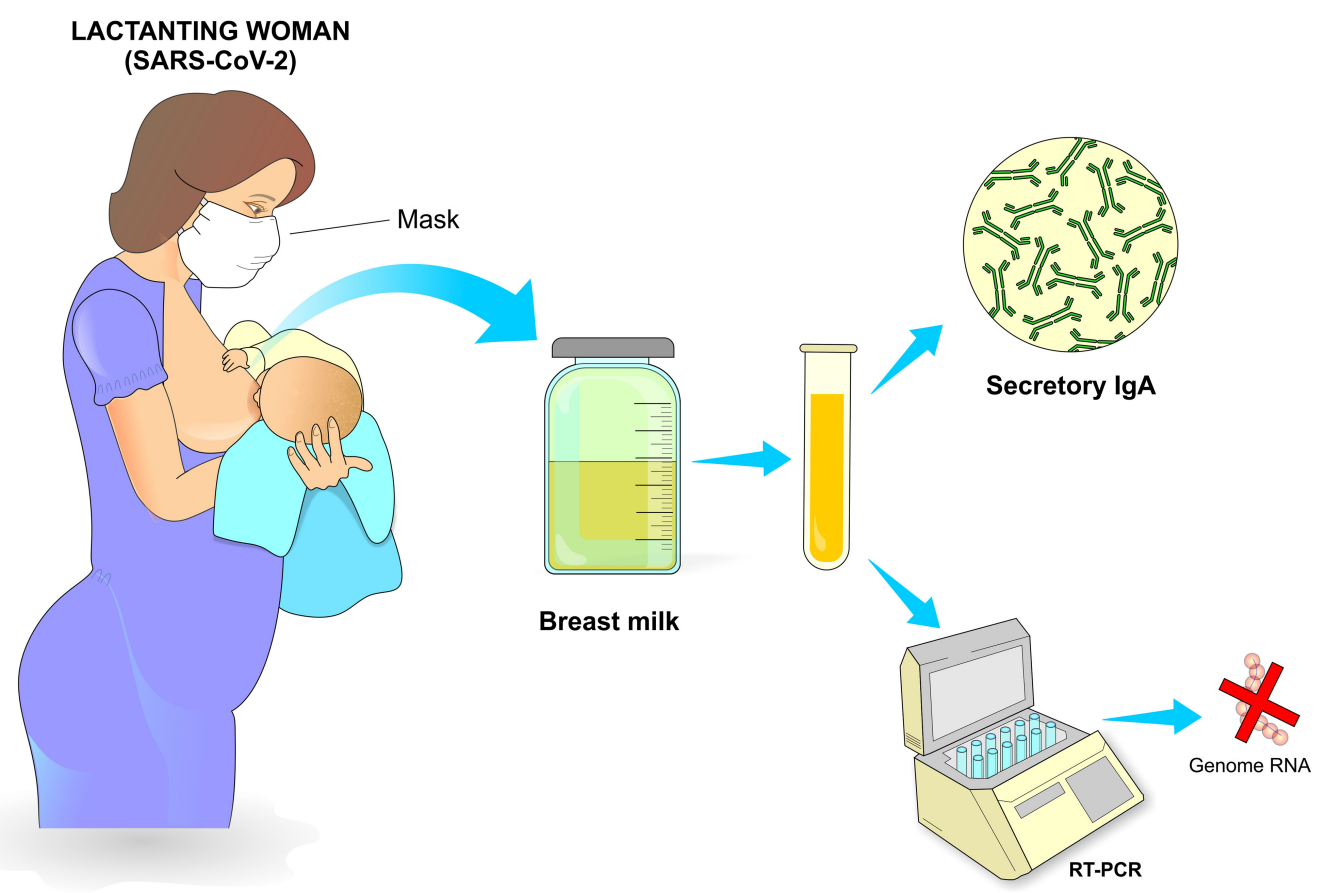
Postpartum care. Breastfeeding is recommended as long as the conditions of the infected mother are appropriate, which include adequate breastfeeding procedure, use of a mask with adequate filtering, cleaning of the breasts and nipples and general care that minimizes contamination of the newborn. Milking can also be carried out, preferably with an appropriate suction pump after previous hygiene of the breasts and with subsequent adequate storage. The presence of secretory IgA in breast milk represents an important passive protection transmitted from the mother to the newborn through breastfeeding.

Breastfeeding in patients infected with SARS-CoV-2 is not contraindicated, as long as they have the desire to breastfeed and have stable clinical conditions. Factors such as severity of the symptoms, hygiene of the breasts, use of mask, and respiratory hygiene must be considered before and during breastfeeding. A study carried out by German researchers evaluated by RT-PCR the presence of the virus in milk samples from 2 infected women. In the four samples collected from one of the mothers, the tests were negative for SARS-CoV-2, whereas the milk collected from the other mother had viral RNA detected for 4 days consecutively. However, the authors claim that more studies need to be carried out to determine whether the virus can be transmitted during breastfeeding (45, 66) (Figure 11).

Hand and Noble state that the anti-inflammatory and anti-infective factors that are present in breastmilk becomes especially important in mitigating infectious conditions, as shown by a recent report that found a strong sIgA antibody SARS-CoV-2 immune response in breastmilk from 12 out of 15 mothers (80%) previously infected with COVID-19 (67).

Mothers infected with SARS-CoV-2 are usually asymptomatic or have mild symptoms. A prospective study investigated 70 pregnant women who had reactive PCR on admission for delivery and of these, 12 (13%) had complications in the puerperium, with 3 being admitted to intensive care unit 7 days after delivery due to hypoxia and tachypnea with signs of multifocal pneumonia and need for oxygen through nasal cannula (54).

One of the first retrospective studies conducted in China reported that between December 2019 and February 2020 nine children aged up to 1 year old were tested positive for SARS-CoV-2. Country data had just reported over 31,000 confirmed cases of Covid-19 in the same period and this study found that at least one member in the family of each child had the infection. In addition, most children had fever and mild respiratory symptoms even though more undiagnosed cases were certainly present in this population, as only hospitalized children were included in the study (68).

A prospective study also conducted in China involving 33 newborns from mothers diagnosed with Covid-19 revealed that only 3 were PCR positive for SARS-CoV-2, two were born at 40 weeks of pregnancy by cesarean sections, which were indicated due to fetal distress and severity of maternal pneumonia. After collection of nasal and rectal swabs, both newborns had the infection confirmed on the second day after birth and presented fever, lethargy and radiological signs of pneumonia. The third child was born by cesarean section at 31 weeks after acute fetal distress and had to be resuscitated. Finally, the latter presented a condition suggestive of neonatal sepsis with positive blood culture for Enterobacter and pneumonia on the chest x-ray (69).

In another prospective study, the authors compared stillbirths, birth weight, Apgar score and number of admissions to the neonatal intensive care unit among newborns from infected (*n* = 69) and non-infected (*n* = 599) women. The results showed no significant difference between these groups with only 1 stillbirth at 37 weeks of gestation from an infected mother with decompensated diabetes (54). In New York, an observational study conducted in 3 hospitals identified 120 neonates born from 116 mothers positive for SARS-CoV-2. All neonates were tested at 24 h of life and none were positive. Eighty-two neonates completed follow-up at day 5–7 of life. All mothers were allowed to breastfeed and 79 of 82 neonates repeated PCR test at 5–7 days of life with negative results in all of them. After 14 days of life, 72 (88%) neonates were also tested and none were positive. None of the neonates had symptoms of COVID-19 (70).

**Figure 12:**
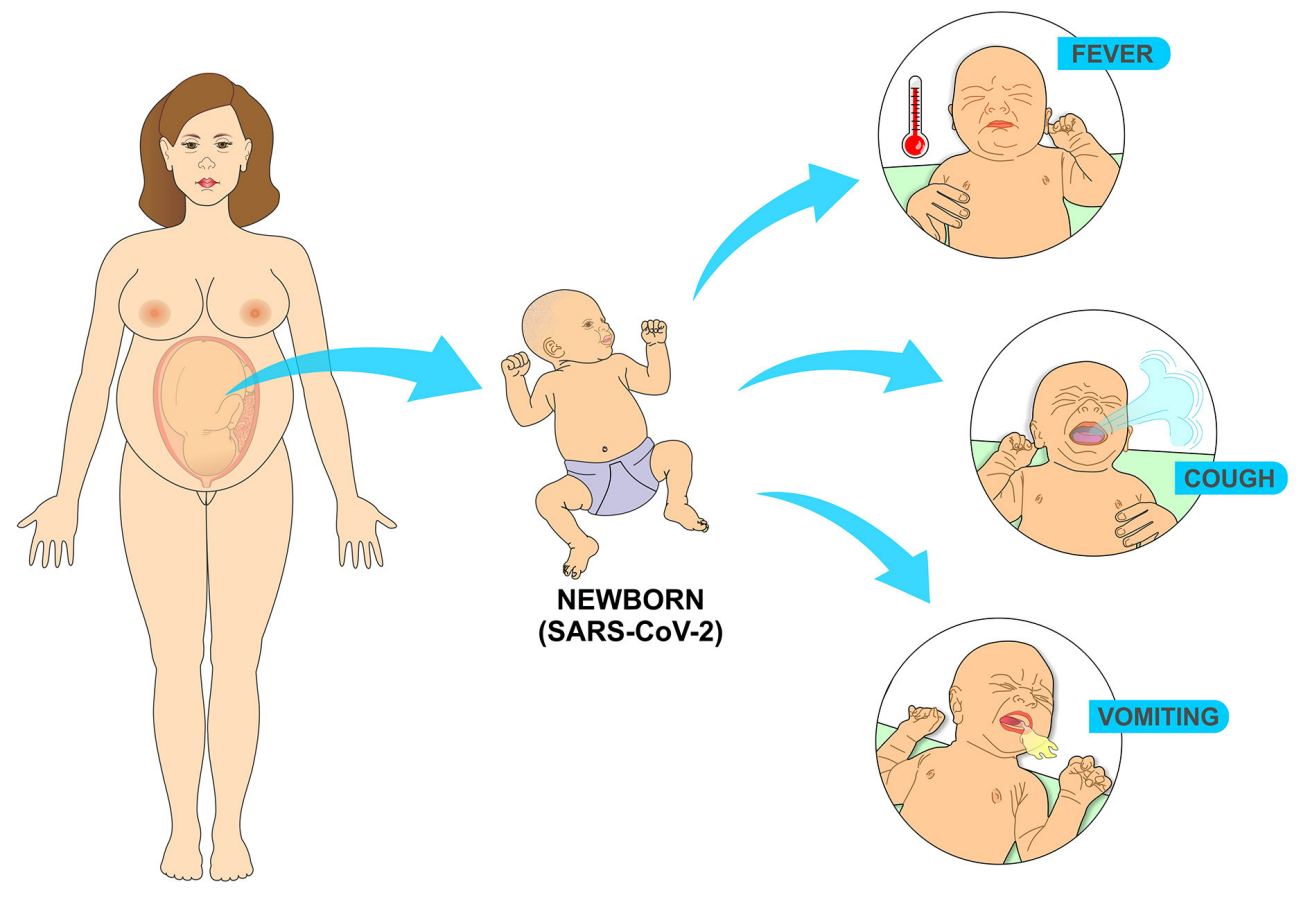
Most common symptoms in the newborn infected with SARS-CoV-2 come from the replication of the virus in the cells of the respiratory tract. Fever results from the immune response to the presence of the virus, while vomiting comes from coughing as a result of the infectious process in the airways.

Literature review investigated the clinical characteristics of 25 neonates with positive RT-PCR in the first 28 days of life. The newborns had an average gestational age of 37 weeks and 4 days and an average birth weight of 3,041 grams. The most common signs and symptoms were fever, vomiting and cough, there were no deaths and the average hospital stay was 15 days, ranging from 5 to 40 days (71) (Figure 12).

## Discussion

In this review, it was found that the increased risk of SARS in pregnant women infected with the new coronavirus can be explained by physiological changes in the respiratory system and by peculiarities in the immune response in this specific population. Studies in the two countries with the highest number of cases of COVID-19, Brazil and the United States, showed higher maternal mortality in the former country and a higher risk of admission to intensive care units in the latter (41, 44).

However, systematic reviews (46–52) that evaluated pregnant women with COVID-19 in different countries reported that the majority were infected in the last two gestational trimesters while presenting the asymptomatic form of the disease or mild symptoms such as fever and cough, with maternal mortality above 1.5% in only one study (51). This fact is probably explained by the average age of infected pregnant women, which does not fit the age group that is mostly affected by the severe form of COVID-19. A review study about the epidemiology of COVID-19 points out that the majority of those infected have mild flu-like symptoms and only 2 to 5% of the cases evolve to SARS (72).

The studies included in this current review showed a higher prevalence of cesarean section and prematurity among pregnant women infected with SARS-CoV-2, which seems to be due to changes in the placental circulation induced by this virus that results in acute or chronic fetal distress and fetal hypoxia. Studies that carried out histopathological analysis of the placentas of pregnant women with the disease have demonstrated a greater presence of thrombi, villous edema, inflammatory infiltrates, poor perfusion in the fetal face and meconium in the placental tissue, which are associated with impaired fetal oxygenation (54, 73).

Vertical transmission of SARS-Cov-2 needs to be proven by studies with a larger sample of infected pregnant women and with a greater number of molecular tests performed on the placenta, amniotic fluid, umbilical cord blood and newborns. In this review, most studies (46–49, 51–53) detected the virus by performing RT-PCR analysis on placental tissue, umbilical cord blood and amniotic cavity. However, the possibility of SARS-Cov-2 being transmitted during pregnancy and childbirth cannot be ruled out as a recent systematic review including fifty studies reported that the virus was detected in newborns as well as in the placentas and amniotic fluid of infected mothers (57).

Likewise, only a single study (64) has detected the presence of the virus in breast milk so far. A review that included eight studies that analyzed the presence of SARS-CoV-2 RNA in the breast milk of 24 pregnant women infected with this virus during the third trimester of pregnancy demonstrated that no breast milk samples were positive for SARS-CoV-2 (74). The World Health Organization (WHO) recommends that women with suspected or confirmed cases of COVID-19 can breastfeed, based on the idea that through breastmilk the babies would get antibodies and anti-infective factors that help protect newborns from getting infections (75). Thus, it seems likely to conclude that there is no restriction for pregnant women with Covid-19 to breastfeed if respecting the recommendations for hand washing and mask use.

In addition, the studies evaluated in this current review indicated that the prevalence of preterm delivery and cesarean sections was high among pregnant women with Covid-19, in which most of them had the asymptomatic or mild form of the disease, with low mortality and with few cases of infected neonates shortly after delivery. In infected newborns, mortality was similarly low, with a mild form of fever and cough, with a low rate of hospitalization. Most studies covered in this review involved pregnant women diagnosed in the last two trimesters of pregnancy, which seems to prevent the infection from being associated with a higher risk of miscarriage. Moreover, research involving neonates had a very short follow up, impairing the assessment of the effects of the virus on children's health in the first year of life. Further studies are needed to prove vertical transmission and to ensure that the virus is not present in the placenta, amniotic fluid, umbilical cord blood and breast milk.

## Author Contributions

AV wrote the topics SARS-Cov-2 in pregnancy, childbirth, and its vertical transmission and SARS-Cov-2 in the postpartum. AF wrote the topic SARS-Cov-2: mechanisms of cell infection and immune response and was responsible for organizing the references and citations. FG wrote the topic Pregnancy, immunology, and susceptibility to SARS-Cov-2. FP was responsible for designing all the figures in this manuscript. EdA was responsible for translating the manuscript to English and for revising the entire manuscript. RC wrote the topic SARS-Cov-2 in pregnancy, childbirth, and its vertical transmission, was responsible for the concept of this work, and also helped writing the abstract, introduction, and discussion sections. All authors contributed to the article and approved the submitted version.

## Conflict of Interest

The authors declare that the research was conducted in the absence of any commercial or financial relationships that could be construed as a potential conflict of interest.
